# Effect of Nitrogen Fertilizer on the Rhizosphere and Endosphere Bacterial Communities of Rice at Different Growth Stages

**DOI:** 10.3390/ijms252413702

**Published:** 2024-12-22

**Authors:** Jinjun Wang, Wang Miao, Shiyu Li, Mingliang Yang, Xinru Gao

**Affiliations:** 1 Key Laboratory of Arable Land Quality Monitoring and Evaluation, Ministry of Agriculture and Rural Affairs, Yangzhou University, Yangzhou 225009, China; 2 College of Environmental Science and Engineering, Yangzhou University, Yangzhou 225127, China; miaowang_0322@163.com (W.M.); shirleyleenn@163.com (S.L.); yasmine0787@163.com (M.Y.); gxr0207@163.com (X.G.)

**Keywords:** growth stage, nitrogen fertilizer, rhizosphere, endosphere, bacterial community

## Abstract

This study aimed to investigate the impact of nitrogen (N) fertilizer on bacterial community composition and diversity in the rhizosphere and endosphere of rice at different growth stages. Two treatments, N0 (no N application) and N1 (270 kg N ha^−1^), were implemented, with samples collected during the jointing, tasseling, and maturity stages. High-throughput sequencing was used to analyze the structure and composition of bacterial communities associated with Huaidao No. 5 (japonica conventional rice). The findings indicated that root zone location was the primary factor influencing the diversity and composition of rice root-associated bacterial communities. Further analysis revealed that nitrogen fertilizer primarily influenced rhizosphere bacterial diversity, while endosphere bacterial diversity was more significantly affected by growth stages. Rice recruited distinct beneficial bacteria in the rhizosphere and endosphere depending on the growth stage. Additionally, the relative abundance of functional genes related to nitrogen metabolism in root-associated bacteria was not significantly influenced by nitrogen application at 270 kg N ha^−1^. These findings offer valuable insights into how nitrogen fertilizer affects plant root bacterial communities across different growth stages.

## 1. Introduction

Plant root microorganisms encompass a diverse array of microbes associated with different regions of the root system, including the rhizoplane, rhizosphere, and endosphere et al. [1,2]. These microorganisms form complex interactions with their host plants [3,4], significantly influencing plant growth, development, nutrient uptake, and disease resistance [5,6,7,8,9]. Rhizosphere microorganisms inhabit the soil surrounding the roots, where they play a crucial role in nutrient cycling. Their metabolic activities, such as respiration and organic acid production, contribute to the dissolution of insoluble minerals, thereby enhancing the availability of phosphorus and other essential nutrients for plant uptake. On the other hand, endosphere microorganisms, residing within the root tissues, influence plant growth and development by secreting bioactive substances, including phytohormones [10]. Current research emphasizes the roles of beneficial root microorganisms, such as nitrogen-fixing bacteria, mycorrhizal fungi, plant growth-promoting rhizobacteria (PGPR), and biocontrol bacteria. These microbes contribute to plant growth directly by supplying nutrients and modulating hormonal pathways or indirectly by enhancing plant immunity and suppressing pathogens [11]. Notably, specific bacterial genera, such as *Variovorax*, have been shown to promote root elongation and even mitigate growth inhibition caused by other microbial strains [12]. Therefore, it is of great practical significance to deeply explore the response of plant root microbial communities to agronomic measures.

Rice, a staple food for nearly half of the world’s population, ranks among the three most important global food crops [13]. Nitrogen (N) is a crucial nutrient for plant growth, significantly influencing the soil microenvironment and crop productivity. N fertilizer application has become a fundamental agronomic practice to boost rice yields, but optimizing its use is critical for achieving a balance between food security and environmental sustainability [14]. Research indicates that N fertilizer profoundly affects the microbial diversity and composition of plant root systems. For instance, Wang et al. [15] reported that rhizosphere bacterial diversity in *Bothriochloa ischaemum* increased with low to moderate N fertilizer application but decreased or plateaued at high N levels. Similarly, Fan et al. [16] found that long-term N addition reduced the diversity of wheat root-associated bacteria. Chen et al. [17] observed that optimized N application in rice rhizospheres decreased specific denitrifying bacteria while increasing nitrifying bacteria, accompanied by higher Shannon, Pielou, and Simpson indices. Additionally, there was an increase in the relative abundance of phyla such as Proteobacteria and Actinobacteria, while Firmicutes and Bacteroidetes declined under efficient N application.

At present, most previous studies have only studied a certain growth stage of a crop or only investigated the response of rhizosphere microorganisms to nitrogen application, ignoring the dynamic changes in microorganisms in the whole growth cycle of the crop and in different regions of the root system. It has been demonstrated that the microbial community composition of a plant root system dynamically adjusts with the stage of plant growth and development, and plants will recruit different rhizosphere microorganisms at different growth stages to meet their moderate functional requirements. Monteiro et al. [18] found that the community structure of nitrogen-fixing bacteria in the rhizosphere changed significantly during the early stage of growth of *Chrysopogon zizanioides* (L.) Roberty, using denaturing gradient gel electrophoresis and stabilizing after 3 months of growth. Chaparro et al. [19] found that the root microbiota of rice at the seedling stage were significantly different from those of other growth stages. Zhang et al. [20] studied the pattern of change in the root microbiome during the entire rice reproductive period and found that the root microbiome changed gradually with the developmental period of rice and began to stabilize after entering the reproductive growth stage. The relative abundance of δ-Proteobacteria in the root increased significantly, whereas the relative abundance of β-Proteobacteria, Firmicutes, and γ-Proteobacteria declined. However, relatively few studies have been conducted on whether changes in nitrogen demand in rice at different growth stages affect the structure of a root microbial community. Therefore, it is important to study the spatial dynamics of a root microbial community and its mechanisms throughout the growth stages of a crop under different N application levels.

The application of nitrogen fertilizer significantly alters the composition, diversity, and functional potential of bacterial communities in the rhizosphere and endosphere of rice plants, with these effects varying across different growth stages due to dynamic shifts in plant nutrient demands, root exudate profiles, and microenvironmental conditions. Specifically, we hypothesize that nitrogen fertilization enhances the relative abundance of nitrogen-cycling bacteria (e.g., nitrifiers and denitrifiers) in the rhizosphere, particularly during vegetative growth stages when nitrogen uptake is highest; induces distinct successional patterns in bacterial communities between the rhizosphere and endosphere across growth stages due to differences in root physiology and nutrient partitioning; and decreases overall microbial diversity in the rhizosphere at later stages of growth due to the potential nutrient imbalances and dominance of specific nitrogen-responsive taxa.

In this study, we employed high-throughput sequencing technology to investigate the bacterial community structure in the rhizosphere and endosphere of rice at different growth stages under varying nitrogen (N) fertilizer levels. Using Huaidao No. 5, a conventional japonica rice variety, as the model plant, we aimed to (1) evaluate the response of rhizosphere and endosphere bacterial community structure and diversity to different N fertilizer treatments and (2) explore the dynamics of these bacterial communities across distinct growth stages of rice. The findings from this research provide a theoretical foundation for enhancing nitrogen utilization efficiency in rice through tailored fertilization strategies. By advancing our understanding of the interplay between rice growth stages, nitrogen management, and root-associated microbiomes, this work contributes valuable insights toward the sustainable development of agroecosystems.

## 2. Results

### 2.1. α-Diversity of Bacterial Communities in the Rhizosphere and Endosphere of Rice

**Figure 1:**
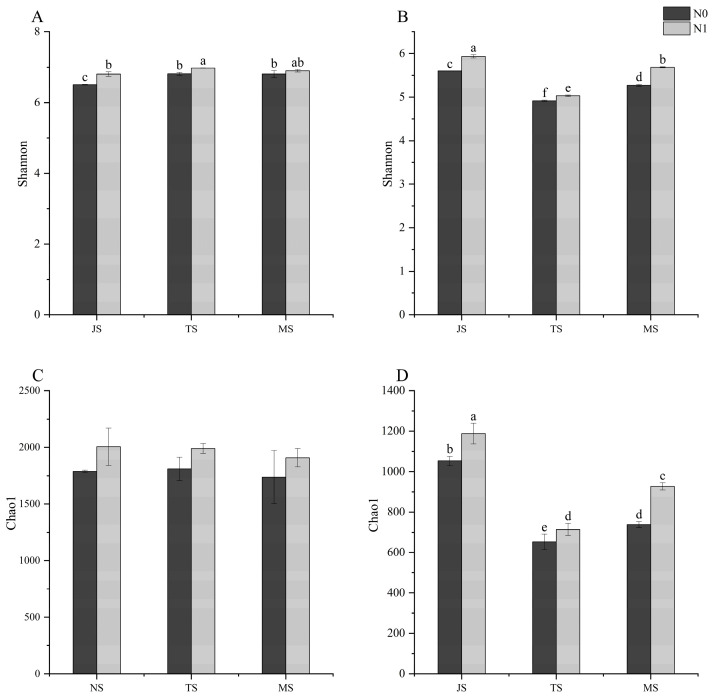
The rhizosphere and endosphere Shannon index and Chao1 index. (**A**,**C**) are the Shannon index and Chao1 index in the rhizosphere, respectively. (**B**,**D**) are the Shannon index and Chao1 index in the endosphere, respectively. Abbreviations: JS, jointing stage; TS, tasseling stage; MS, maturity stage. Different letters represent significant differences at *p* < 0.05, respectively.

The Chao1 index in the rhizosphere did not conform to a normal distribution, and the Kruskal–Wallis test of nonparametric tests was used, resulting in no significant differences between any of the treatments (Figure 1C). The rhizosphere’s Shannon index was significantly higher in the N1 treatment than in the N0 treatment at both JS and TS, and there was no significant difference at MS (*p* < 0.05). Regarding growth stages, there was a significant difference between the JS and TS Shannon indices, which were not significantly different from MS (Figure 1A). As for the endosphere, both the Shannon and Chao1 indices showed a tendency of decreasing and then increasing as the growth stage progressed, and the N1 treatment was significantly higher than the N0 treatment (Figure 1B,D).

### 2.2. β-Diversity of Bacterial Communities in the Rhizosphere and Endosphere of Rice

**Figure 2:**
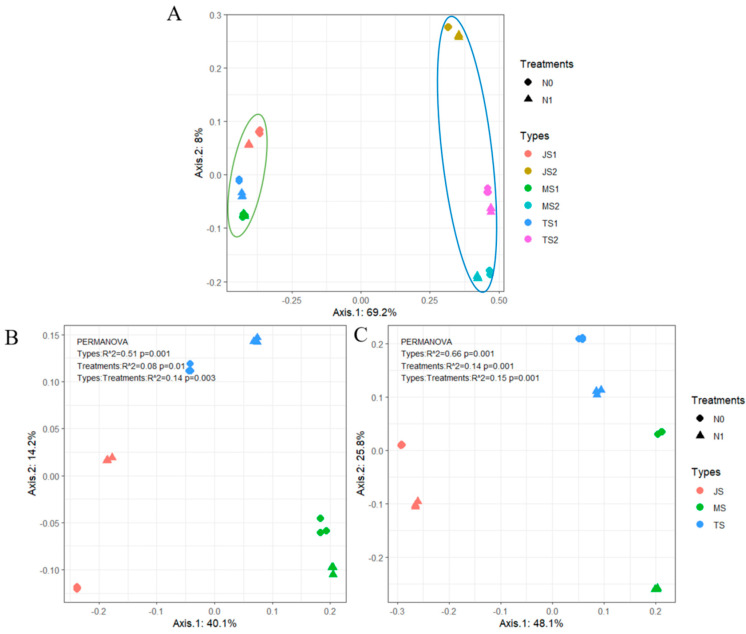
Principal coordinate analysis (PCoA) of root bacterial communities. (**A**) PCoA of the rhizosphere and endosphere bacterial communities across all treatments, where 1 represents rhizosphere and 2 represents endosphere; (**B**) PCoA of the rhizosphere bacterial community; (**C**) PCoA of the endosphere bacterial community.

PCoA was used to visualize the differences in the bacterial communities (Figure 2). Based on the location of the root zone, the rhizosphere and endosphere bacterial communities were divided into two distinct clusters, in which the rhizosphere bacterial communities were clustered closer together and the endosphere bacterial communities were clustered farther apart. This suggests that the location of the root zone is the most important factor influencing a bacterial community. Figure 2B,C revealed significant differences in bacterial communities at different growth stages of the rhizosphere and endosphere. The PERMANOVA results showed that the growth stage was the main factor in bacterial community variation, explaining 51.4% and 65.9% of the variation in rhizosphere and endosphere bacterial communities (Table 1).

### 2.3. Compositions of Rhizosphere and Endosphere Bacterial Communities

**Figure 3:**
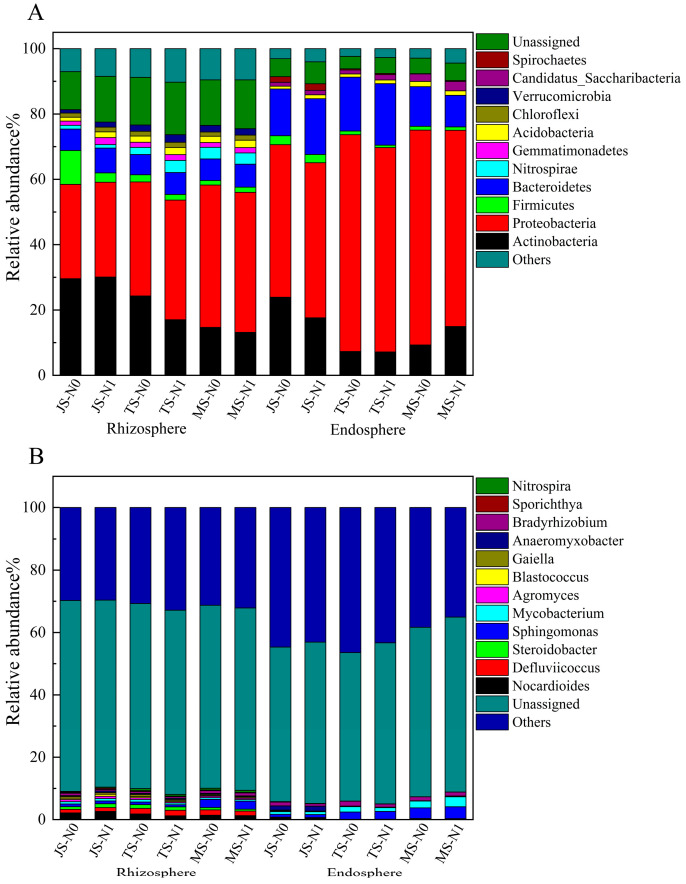
Composition of rhizosphere and endosphere bacterial communities under different treatments. (**A**) Relative abundance of the dominant phyla. (**B**) Relative abundance of the dominant genus.

**Figure 4:**
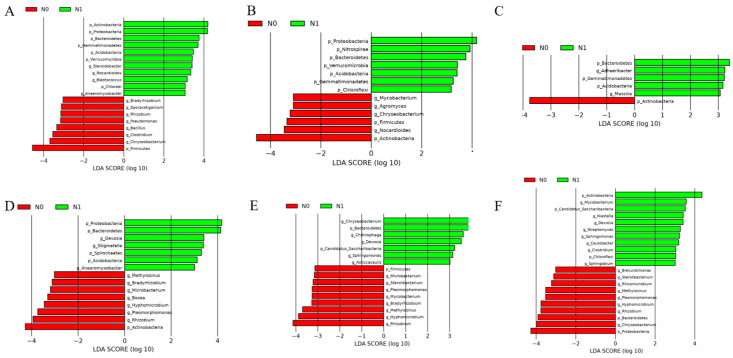
Linear discriminant analysis (LDA) of bacterial community composition in the rhizosphere and endosphere of rice at different growth stages. (**A**–**C**) represent JS, TS, and MS in the rhizosphere, respectively. (**D**–**F**) represent JS, TS, and MS in the endosphere, respectively. Only the differences with a logarithmic LDA score > 3 and *p* < 0.05 are presented.

Actinobacteria, Proteobacteria, Firmicutes, Bacteroidetes, Nitrospirae, Gemmatimonadetes, Acidobacteria, and Chloroflexi were the dominant phyla in the individual treatments of the rhizosphere. Actinobacteria, Proteobacteria, Firmicutes, Bacteroidetes, Acidobacteria, Candidatus_Saccharibacteria, and Spirochaetes were the dominant phyla in the individual treatments of the endosphere (Figure 3A). The LDA results for the rhizosphere and endosphere bacterial community compositions under the N1 and N0 treatments across three growth stages were as follows. In the rhizosphere, at the jointing stage, the N1 treatment significantly enriched Actinobacteria, Proteobacteria, Bacteroidetes, Gemmatimonadetes, Acidobacteria, Verrucomicrobia, *Steroidobacter, Nocardioides, Blastococcus,* Chlorobi, and *Anaeromyxobacter*. Conversely, the N0 treatment was enriched in *Bradyrhizobium, Sporacetigenium, Rhizobium, Pseudomonas, Bacillus, Clostridium, Chryseobacterium*, and Firmicutes (Figure 4A). At the tasseling stage, N1 was enriched in Proteobacteria, Nitrospirae, Bacteroidetes, Verrucomicrobia, Acidobacteria, Gemmatimonadetes, and Chloroflexi, while N0 showed enrichment in *Mycobacterium, Agromyces, Chryseobacterium,* Firmicutes, *Nocardioides,* and Actinobacteria (Figure 4B). At the maturity stage, N1 was enriched in Bacteroidetes, *Adhaeribacter*, Gemmatimonadetes, Acidobacteria, and *Massilia*. N0, on the other hand, was significantly enriched in Actinobacteria (Figure 4C).

In the endosphere, at the jointing stage, N1 treatment led to enrichment in Proteobacteria, Bacteroidetes, *Devosia, Stigmatella,* Spirochaetes, Acidobacteria, and *Anaeromyxobacter*, while N0 was enriched in *Methylosinus, Bradyrhizobium, Microbacterium, Bosea, Hyphomicrobium, Pleomorphomonas, Rhizobium*, and Actinobacteria (Figure 4D). At the tasseling stage, N1 was enriched with *Chryseobacterium*, Bacteroidetes, *Chitinophaga, Devosia*, Candidatus_Saccharibacteria, *Sphingomonas,* and *Asticcacaulis*. N0 treatment favored Firmicutes, *Microbacterium, Sterolibacterium, Pleomorphomonas, Mycobacterium, Bradyrhizobium, Methylosinus, Hyphomicrobium*, and *Rhizobium* (Figure 4E). At the maturity stage, N1 treatment enriched Actinobacteria, *Mycobacterium*, Candidatus_Saccharibacteria, *Niastella, Devosia, Streptomyces*, *Sphingomonas*, *Caulobacter*, *Clostridium*, Chloroflexi, and *Sphingobium*, whereas N0 treatment enriched *Brevundimonas, Sterolibacterium, Rhizomicrobium, Methylosinus, Pleomorphomonas*, *Hyphomicrobium*, *Rhizobium,* Bacteroidetes, *Chryseobacterium*, and Proteobacteria (Figure 4F). These results illustrate the distinct bacterial taxa recruited by rice in the rhizosphere and endosphere at different growth stages, with compositions varying significantly between the N1 and N0 treatments, highlighting the impact of nitrogen fertilization on microbial community assembly.

### 2.4. Effects on the Abundance of Functional Genes for Nitrogen Metabolism

**Figure 5:**
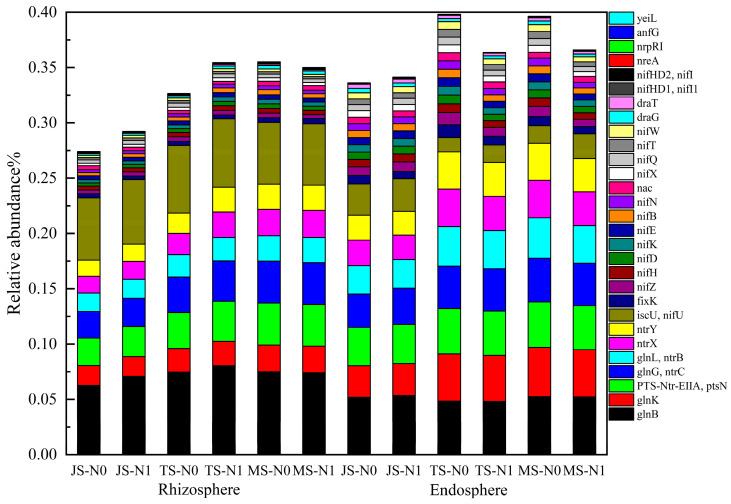
Relative abundance of functional genes related to nitrogen metabolism in the rhizosphere and endosphere.

N1 treatment significantly enhanced the total abundance of JS and TS functional genes in the rhizosphere, but significantly reduced the total abundance of TS and MSfunctional genes in the endosphere (Figure 5). LDA analysis showed that the N1 treatment significantly altered the abundance of individual nitrogen metabolism genes in both the rhizosphere and endosphere. The endosphere exhibited a higher abundance of these functional genes compared to the rhizosphere as the growth stage progressed.

**Figure 6:**
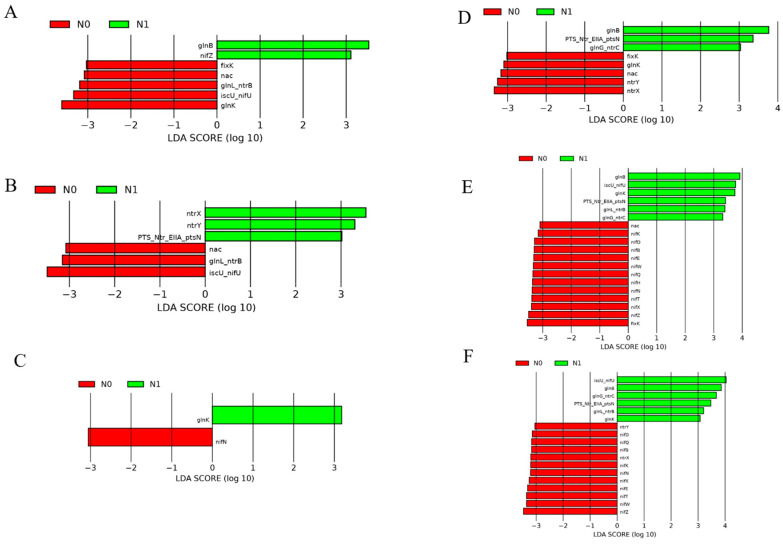
Linear discriminant analysis (LDA) of functional genes related to nitrogen metabolism in the rhizosphere and endosphere of rice at different growth stages. (**A**–**C**) represent JS, TS, and MS in the rhizosphere, respectively. (**D**–**F**) represent JS, TS, and MS in the endosphere, respectively. Only the differences with a logarithmic LDA score > 3 and *p* < 0.05 are presented.

In the rhizosphere, at the jointing stage, N1 significantly enriched *glnB* and *nifZ*, while N0 enriched *fixK, nac, ntrB, nifU*, and *glnK.* At the tasseling stage, N1 was enriched in *ntrX, ntrY*, and *ptsN*, while N0 was enriched in *nac, ntrB, and nifU*. At the maturity stage, N1 was enriched in *glnK*, and N0 in *nifN*. In the endosphere, at the jointing stage, N1 treatment significantly enriched *glnB, ptsN*, and *ntrC*, while N0 enriched *fixK, glnK, nac, ntrY,* and *ntrX*. At the tasseling stage, N1 showed significant enrichment in *glnB, nifU, glnK, ptsN, ntrB,* and *ntrC*, whereas N0 was enriched in a broad range of nitrogen fixation genes, including *nac, nifK, nifD, nifB, nifE, nifW, nifQ, nifH, nifN, nifT, nifX, nifZ,* and *nixK*. At the maturity stage, N1 was enriched in *nifU, glnB, ntrC, ptsN, ntrB,* and *glnK*, while N0 was enriched in *ntrY, nifD, nifQ, nifB, ntrX, nifK, nifN, nifX, nifE, nifT, nifW,* and *nifZ*. These results indicate that the N1 and N0 treatments differentially affected specific nitrogen metabolism genes across growth stages, particularly in the endosphere, where the abundance of functional genes was higher compared to the rhizosphere (LDA > 3, *p* < 0.05) (Figure 6).

## 3. Discussion

### 3.1. The Growth Stage Is the Primary Factor Affecting Rhizosphere and Endosphere Bacterial Communities

The composition and diversity of microbial communities in rice roots differ significantly between spatial structures, with the root zone location being a key factor in shaping the bacterial diversity of Huaidao No. 5. The rhizosphere’s bacterial diversity is consistently higher than that of the endosphere. This disparity is largely due to the “gate valve” role played by a root surface, which selectively enriches bacterial taxa from the rhizosphere into the endosphere, resulting in a distinct microbial community structure between these two regions [21]. These findings align with those reported by Jiang et al. [22], Monteiro et al. [23], and Xu et al. [24]. Diversity metrics, such as the Shannon and Chao1 indices, followed a trend of decreasing and then increasing as the growth stage progressed (Figure 1B,D). The rhizosphere, being more dynamic and directly exposed to a soil environment, shows rapid responses to nitrogen (N) application due to shifts in its chemistry, including nitrogen, phosphate, and organic matter levels. This exposure leads to a quicker response of sensitive species in the rhizosphere community to N inputs [25,26,27]. In contrast, the endosphere provides a relatively isolated and stabilized environment, making it less sensitive to external environmental changes. Rhizosphere bacteria, being directly connected to external soil, have greater access to nutrients and are generally more adaptable. While nitrogen application increases available nitrogen sources for rhizosphere bacteria, endosphere bacteria primarily rely on nitrogen assimilated by the host plant, resulting in a comparatively weaker response to nitrogen inputs [28,29].

The PCoA analysis results highlighted root zone location as the most significant factor influencing the rice root bacterial community structure (Figure 2A). When examining the effects of nitrogen application and growth stage on rhizosphere and endosphere communities, PERMANOVA analysis further identified growth stage as the predominant factor in structuring these bacterial communities (Table 1), with notable shifts occurring as the growth stage advanced. These findings align with studies by Edwards et al. [4] and Zhang et al. [20], who observed similar patterns of microbiome dynamics across the reproductive period in rice. Early in plant growth, root secretions attract a large influx of soil microorganisms to the rhizosphere, with some colonizing the plant interior. This leads to high variability in the microbial community during the plant’s nutrient growth stage. However, as rice enters the reproductive growth stage, the rhizosphere microbial community stabilizes, likely due to fewer root exudate-induced fluctuations [30].

In Huaidao No. 5 rice, Actinobacteria, Proteobacteria, Firmicutes, Bacteroidetes, and Acidobacteria were identified as dominant phyla in both the rhizosphere and endosphere. This finding is consistent with previous studies on the rice rhizosphere microbiome [1,24,31]. Proteobacteria, Bacteroidetes, and Firmicutes are generally regarded as eutrophic taxa, capable of rapid growth in environments enriched with labile carbon [32,33]. The LDA results indicated that rice selectively recruits various beneficial bacterial genera at different growth stages (Figure 4). Key genera such as *Devosia, Anaeromyxobacter*, and *Clostridium* possess nitrogen-fixing capabilities and play vital roles in nitrogen cycling within soil and root systems [34,35,36]. Other genera, including *Devosia, Massilia, Chryseobacterium, Sphingomonas, Streptomyces, Caulobacter, Asticcacaulis,* and *Sphingobium*, are known as plant growth-promoting (PGPR) rhizobacteria [37,38,39,40,41]. These PGPR rhizobacteria enhance plant growth and nutrient uptake and boost stress tolerance through mechanisms like nitrogen fixation, phosphorus solubilization, antibiotic production, and phytohormone secretion. As the rice growth stage progresses, the biomarker type and relative abundance of microbes in the rhizosphere and endosphere also shift significantly. Notably, *Devosia* emerged as a persistent biomarker across growth stages in the endosphere under N1 treatment. This genus, classified as α-Proteobacteria, effectively colonizes plant roots and exhibits multifunctional plant growth-promoting attributes. *Devosia* harbors nitrogen-fixing genes, like nifH, and secretes phytohormones (e.g., indoleacetic acid, IAA), solubilizes minerals like phosphorus, and supports overall plant growth through diverse mechanisms [34]. In summary, as rice enters different stages of its reproductive period, its root-associated microbiota dynamically shift to recruit beneficial bacteria, thereby maintaining a supportive root environment and promoting plant health and growth.

### 3.2. Functional Gene Abundance of Nitrogen Metabolism at Different Growth Stages in Response to Nitrogen Fertilization

Functional genes involved in nitrogen metabolism in agricultural soils provide critical insights into the species and abundance of functional microorganisms that drive specific nitrogen transformation pathways [42]. The results of LDA analysis showed that the types or abundances of functional genes for nitrogen metabolism were significantly higher in the endosphere than the rhizosphere as the growth stage progressed, and this difference was particularly significant at maturity. This may be because the heightened demand for nutrients in mature rice plants spurs active nitrogen metabolism within the roots, while the enclosed endosphere environment intensifies the expression of nitrogen metabolism genes. Conversely, rhizosphere microorganisms may compete with or regulate nitrogen utilization within the roots, leading to a differential effect on gene abundance [43,44].

Among these functional genes, *glnB* and *ptsN* emerged as biomarkers throughout the entire growth stage in the endosphere. The gene *glnB*, which encodes the PII protein, displayed the highest relative abundance across treatments. PII proteins play an essential role in sensing intracellular nitrogen levels (by monitoring metabolites like α-ketoglutarate, ATP, and ADP) and respond by initiating nitrogen fixation under low-nitrogen conditions, thereby enabling bacteria to utilize atmospheric nitrogen [45,46]. The gene *ptsN* encodes the EIIA (*Ntr*) protein, which is also critical for nitrogen metabolism and interacts with PII proteins from *glnB* to regulate nitrogen-related gene expression [47]. Notably, the relative abundance of *ptsN* remained fairly stable across growth stages but accounted for a large proportion of all functional genes (Figure 5). The results of this study align with previous research, such as that by Xu et al. [48], who reported that nitrogen application did not significantly impact the relative abundance of the nitrogen-fixing gene *nifH*. In this study, nitrogen application at 270 kg N ha^−1^ similarly showed no substantial effect on the abundance of nitrogen metabolism genes, though the growth stage did influence the types and abundances of these genes. By contrast, Li et al. [49] and Li et al. [50] reported an increase in *nifH* expression with nitrogen addition. These differing findings may be attributed to background nitrogen levels in the soil; when nitrogen-fixing bacteria can readily absorb soil nitrogen, their need to fix atmospheric nitrogen decreases, leading to the downregulated expression of nitrogen fixation genes [51]. Moreover, nitrogen application alters the composition of rhizosphere microbial communities, and external factors such as temperature, moisture, and soil conditions further influence nitrogen utilization efficiency [52]. These environmental variables impact the structure of rhizosphere microbial communities and, consequently, the expression of nitrogen metabolism-related genes, highlighting the complex interplay between the growth stage, soil conditions, and microbial nitrogen cycling in rice.

## 4. Materials and Methods

### 4.1. Experimental Design

The field experiment was conducted in ShantouTown, Yangzhou City, Jiangsu Province (119°52′N, 32°31′E), using the rice variety Huaidao No. 5. Two nitrogen (N) application treatments were established as the following: no N application (N0) and conventional N application (N1, 270 kg N ha^−1^). The experiment utilized a randomized block design with three replicates per treatment. Each plot measured 9 m^2^ (3 m × 3 m), with a transplanting density of 30 cm × 16 cm, and two seedlings per hill. In addition to nitrogen, phosphate fertilizer (P_2_O_5_, 12%) at 1000 kg ha^−1^ and potash fertilizer (K_2_O, 60%) at 200 kg ha^−1^ were uniformly applied across all plots. The experimental field was characterized by conventional soil with the following baseline physicochemical properties: pH 7.42, ammonium nitrogen (NH_4_^+^) 10.7 mg/kg, nitrate nitrogen (NO_3_^−^) 9.53 mg/kg, total nitrogen (TN) 1.57 g/kg, organic matter (OM) 51.02 g/kg, and electrical conductivity (EC) 0.4 mS/cm. Other field management practices, such as weeding and pest control, were performed according to standard high-yield cultivation techniques.

### 4.2. Rice Root Sample and Rhizosphere Soil Sample Collection

Rice rhizosphere soil was collected according to the method described by Edwards et al. [1]. Roots with 1 mm soil attached were placed in a triangular vial containing 50 mL of sterile phosphate buffer and vortexed for 10 min to clean all soil from the root surface. The soil cleaned from the roots was freeze-dried and stored as rhizosphere soil in a −80 °C freezer. Root surface decontamination of the above washed rice roots was performed with reference to the method described by Wang et al. [53]. First, washed rice roots were placed in sterile 50 mL centrifuge tubes, and 30 mL of sterile phosphate buffer (containing 0.02% Tween 20) was added to submerge the samples. The samples were processed in an ultrasonic cleaner (SB-5200) at 40 kHz for 1 min, and the procedure was repeated five times, followed by a 5 min immersion in 2% sodium hypochlorite solution, then washing again with sterile water. The final washing solution was used as a template for PCR amplification to check whether surface sterilization was complete. After surface sterilization, the plant samples were lyophilized, pulverized, sieved, and stored at −80° C until DNA extraction.

### 4.3. DNA Extraction and Illumina NovaSeq Sequencing

Root and soil samples were extracted using the FastDNA^®^SPIN Kit for soil (MP Biomedicals, Santa Ana, CA, USA), which was described in the kit manual. The DNA of the samples was detected by 2% agarose gel electrophoresis and stored at −20 °C for subsequent analysis. Primers 799F (5′-AACMGGATTAGATACCCKG-3′) and 1193R (5′-ACGTCATCCCCACCTTCC-3′), which contained index sequences, were used to amplify the V5–V7 region of the 16S rRNA gene of soil and root bacteria [54]. PCR amplification products were purified and sent to Shanghai Tianhao Biotechnology Co., Ltd. (Shanghai, China), for sequencing using the NovaSeq 6000 platform (Illumina, San Diego, CA, USA) with the bipartite sequencing strategy of SP-Xp (PE250).

Raw sequencing data were processed using QIIME2 software (2023.5) [55]. Firstly, the primer fragments of the sequences were excised and the unmatched primers were removed. Then, according to the default settings, the DADA2 plug-in was invoked to perform quality control, denoising, splicing [56], and chimera removal on the sequences to form the characteristic sequences of Amplicon Sequence Variants (ASVs) and the ASV abundance table. Species annotation was performed using a Naive Bayes classifier pre-trained on the Silva132 database, according to default parameters (confidence level 0.8). To correct for differences in bacterial community diversity due to sequencing depth, a random leveling process was performed for each sample. The leveling depth was based on the lowest sequence count in the sequenced samples.

### 4.4. Statistical Analysis

The data were processed using Microsoft Excel 2016, and the bacterial Chao1 index and Shannon indices were calculated using QIIME2. IBM SPSS Statistics 24 was used to test the significance of the Shannon index, Chao1 index, relative abundance of bacterial composition of different treatments, as well as the effect of root zone location, nitrogen application, and fertility stage, on the relative abundances of the bacterial Shannon index, Chao1 index, and bacterial composition, and the difference was statistically significant at *p* < 0.05. Principal coordinate analysis (PCoA) based on the relative abundance of ASVs was performed using the “vegan” package in R4.1.1 to visualize the differences in the rhizosphere and endosphere community structure. Permutational multivariate ANOVA (PERMANOVA) based on Bray–Curtis distance matrices was performed with 999 permutations using the “vegan” package in R4.1.1 to quantify the contribution of growth stages and N fertilization to community variation. Linear discriminant analysis (LDA) was performed using ImageGP [57] to elucidate the biomarkers with a logarithmic LDA score > 3 and *p* < 0.05 in bacterial communities.

## 5. Conclusions

In this study, we analyzed the structure and diversity of rhizosphere and endosphere bacterial communities in Huaidao No. 5 under varying nitrogen application rates and growth stages, exploring the effects on the abundances of functional genes associated with nitrogen metabolism. Root zone location was identified as the key factor influencing bacterial diversity and composition in the rice root, with nitrogen application being the primary factor affecting rhizosphere bacterial diversity, while the growth stage more significantly impacted endosphere bacterial community diversity. Additionally, the growth stage was the main driver of variation in both the rhizosphere and endosphere bacterial communities in Huaidao No. 5. The dominant bacterial phyla in both the rhizosphere and endosphere of Huaidao No. 5 were Actinobacteria, Proteobacteria, Firmicutes, Bacteroidetes, and Acidobacteria. Rice at different growth stages recruited distinct beneficial bacteria within the root, which varied by stage. The relative abundance of nitrogen metabolism functional genes in the rhizosphere and endosphere was not significantly affected by nitrogen application at 270 kg N ha^−1^. However, the types and relative abundances of these genes changed as the growth stage progressed, with *glnB, glnK, ptsN, ntrBC,* and *ntrXY* showing higher relative abundances, playing essential roles in regulating nitrogen metabolism at various growth stages. These results are valuable for furthering our understanding of the interactions among a plant growth stage, nitrogen fertilizer application, and root microbiome.

Future research directions should involve selecting different rice varieties, setting different nitrogen application rates, and sampling them at whole growth stages. Using metagenomics and metaproteomics to explore functional shifts in microbial communities under different nitrogen regimes could help identify key genes and pathways involved in nitrogen fixation, nitrification, and denitrification. Functional analysis may also reveal potential biomarkers for nitrogen use efficiency (NUE) in rice systems.

## Figures and Tables

**Table 1:** Contributions of different factors to the bacterial community.

Root Region	Factor	R^2^ (%)	*p*
Rhizosphere	Growth stage	51.4%	0.001 ***
	Nitrogen fertilizer	7.6%	0.01 **
	Growth stage * nitrogen fertilizer	14.3%	0.003 **
Endosphere	Growth stage	65.9%	0.001 ***
	Nitrogen fertilizer	14.0%	0.001 ***
	Growth stage * nitrogen fertilizer	14.5%	0.001 ***

Abbreviations: *,*p* < 0.05; **, *p* < 0.01; ***, *p* < 0.001.

## Data Availability

The data that support the findings of this study are available from the corresponding author upon reasonable request.
